# Coagulation complications following trauma

**DOI:** 10.1186/s40779-016-0105-2

**Published:** 2016-11-22

**Authors:** Wenjun Z. Martini

**Affiliations:** U.S. Army Institute of Surgical Research, 3698 Chambers Pass, JBSA-Fort Sam Houston, Houston, TX 78234-6315 USA

**Keywords:** Traumatic injury, Coagulation, Sepsis, Lethal triad, Pathophysiology

## Abstract

Traumatic injury is one of the leading causes of death, with uncontrolled hemorrhage from coagulation dysfunction as one of the main potentially preventable causes of the mortality. Hypothermia, acidosis, and resuscitative hemodilution have been considered as the significant contributors to coagulation manifestations following trauma, known as the lethal triad. Over the past decade, clinical observations showed that coagulopathy may be present as early as hospital admission in some severely injured trauma patients. The hemostatic dysfunction is associated with higher blood transfusion requirements, longer hospital stay, and higher mortality. The recognition of this early coagulopathy has initiated tremendous interest and effort in the trauma community to expand our understanding of the underlying pathophysiology and improve clinical treatments. This review discusses the current knowledge of coagulation complications following trauma.

## Background

Traumatic injury remains one of the leading causes of death, accounting for about 40% of prehospital death [1, 2]. Uncontrolled hemorrhage from coagulation dysfunction is one of the main potentially preventable causes of the mortality in both civilian and military settings [3–7]. Hypothermia, acidosis, and resuscitative hemodilution have been considered as the significant contributors to coagulation dysfunction after trauma. Over the past decade, clinical observations around the globe have independently shown that coagulopathy may be present as early as hospital admission in some trauma patients. The hemostatic manifestation is associated with increased blood transfusion requirements, longer hospital stay, and higher mortality [7–11]. The recognition of this early coagulopathy leads to the use of new terminology and proposed hypotheses [8, 12, 13]. However, to date, our understanding of the underlying mechanisms remains incomplete. This review summarizes the current knowledge of coagulation complications following trauma.

## Coagulation process

Blood coagulation is an important physiological process, including a series of physical, biochemical and cellular responses after various stimuli. The essence of the process is the production of fibrin clots from fibrinogen (factor I), and thrombin plays a central role catalyzing the reaction [14]. Biochemically, blood clotting is initiated *via* the intrinsic and/or extrinsic pathways. The two pathways converge to form a common pathway to generate thrombin. The intrinsic pathway, or contact activation pathway, consists of the activations of factor VIII, IX, X, XI, XII and Xa complex, resulting in thrombin generation from precursor prothrombin (factor II). The extrinsic pathway is triggered by plasma factor VIIa binding with tissue factor (factor III) released from the injury sites. The factor VIIa/tissue factor complex, once produced, activates additional factor VII, initial thrombin, factor Xa complex, and platelets, resulting in the exponential thrombin burst for rapid clot formation [15]. This classic cascade model provides a biochemical description of the coagulation process and the basis for clinical assessments of coagulation; but it is now considered incomplete because it does not take into consideration the coagulation inhibition in plasma. Clinical standard plasma tests of prothrombin time (PT) and activated partial thromboplastin time (aPTT) reflect the overall enzyme activities involved in the extrinsic and intrinsic pathways, respectively.

The formation of fibrin clots is counterbalanced by its inhibitory and anti-coagulation processes. Circulating antithrombin III inhibits factor Xa and thrombin, with 2000-fold amplified effects by heparin [16]. Tissue factor pathway inhibitor inhibits factor Xa and eliminates the contribution of the extrinsic pathway to clot formation. Activated protein C, the product of the thrombomodulin-thrombin complex, inactivates prothrombinase and the intrinsic pathway [17]. In addition, fibrin clots, once formed, are subject to fibrinolysis by plasmin. Plasmin is generated from inactive protein plasminogen *via* tissue-type plasminogen activator (tPA) [18]. The activity of tPA can be inhibited by plasminogen activator inhibitors (PAI) [19, 20]. The fibrinolytic system is regulated through the generation of plasmin from the activities of tPA, PAI and an antiplasmin inhibitor. Under any normal physiological state, the blood coagulation status is a dynamic process and is the balance of clot formation, anti-coagulation and fibrinolysis.

Another description of the coagulation process is a cell-based model of hemostasis [21]. This model considers the process as three overlapping phases: initiation, amplification and propagation. All three phases are regulated by the properties of cell surfaces, receptors, and coagulation proteins. This model provides the basis of viscoelastic tests, such as thromboelastography (TEG) and rotational thromboelastometry (ROTEM), to profile the dynamic nature of the clotting process and guide resuscitation practice [22, 23].

## Coagulation tests

Early traumatic coagulopathy has been defined by different measurements, including standard plasma tests of PT, aPTT, thrombin time, platelet counts, fibrinogen levels, and blood viscoelastic tests of clotting amplitudes and clot lysis [8, 22, 24–28]. At present,, there is no standard or globally accepted assay for diagnosing early traumatic coagulopathy, although prolonged PT has been used by many investigators to study trauma-induced coagulopathy.

Compared to plasma PT and aPTT, TEG and ROTEM provide more comprehensive description of coagulation status, including measurements of clotting formation time, clotting speed, clot strength, and fibrinolysis. This advantage has made its increased use in the diagnosis of trauma-induced coagulopathy, the prediction of massive transfusion and for guiding the transfusion of blood products [23, 29]. However, TEG and ROTEM have limited sensitivity in reflecting platelet dysfunction and moderate fibrinolysis [23, 30, 31]. A randomized controlled trial is warranted to validate TEG or ROTEM’s role in guiding massive transfusion protocols in trauma patients.

## Coagulation complications after trauma

After traumatic injury, coagulation, anti-coagulation and fibrinolysis are disproportionally affected, leading to impaired hemostasis. The alterations have been found to be dynamic and multifactorial. For simplicity, it is helpful to describe the changes in three phases: 1) acute post-trauma phase, which occurs shortly, within hours, after trauma injury; 2) resuscitation phase, which occurs 24–48 h post trauma, when various resuscitation fluids may be used; and 3) later phase, which occurs days after trauma injury.

### Acute post-trauma phase

Trauma-related coagulopathy has been considered to be primarily due to blood loss from injury, hemodilution from aggressive resuscitation, and the development of hypothermia and acidosis [32, 33]. During the last decade, clinical studies have shown that prolonged PT and aPTT prothrombin time were observed in some trauma patients at emergency room admission [8–10]. This hemostatic complication is independently associated with increased blood transfusion requirement and higher mortality than those with similar injury but without coagulopathy [8–10]. The recognition of this early coagulopathy prior to fluid resuscitation has initiated tremendous interest and effort to expand our understanding of trauma-related coagulopathy. As the results, new terminology has been created to describe the early developed coagulopathy, such as acute coagulopathy of trauma (ACT), acute traumatic coagulopathy (ATC), trauma induced coagulopathy (TIC), and early coagulopathy of trauma. Hypotheses have also been proposed to attempt to explain the underlying mechanisms.

One hypothesis is consumptive coagulopathy, a phenotypic variation of classic disseminated intravascular coagulation (DIC) [34]. Immediately after trauma, trauma injury exposes tissue factor, which is normally present inside tissues, to the circulation and initiates thrombin generation and clot formation. Platelets are activated through a network of regulated interconnecting cellular signals, including collagen in the sub-endothelial matrix binding to glycoprotein VI, von Willebrand Factor (vWF) and glycoprotein Ib [15]. The activation of platelets amplifies thrombin generation and the clotting process, causing the consumption of the coagulation factors. The most depleted factors are fibrinogen and factor V [35]. In addition, fibrinolysis is activated from the release of tissue plasminogen activator, which converts plasminogen to plasmin, into the circulation. Consequently, hypocoagulation and hyperfibrinolysis are developed in trauma patients.

Another hypothesis considers that activated protein C plays a central role in enhancing anti-coagulation [8, 24, 36, 37]. Based on this hypothesis, following severe trauma injury and hypoperfusion, thrombin is generated and binds thrombomodulin to form activated protein C. Activated protein C exerts its anticoagulant role by inhibiting factor Va and VIIIa and its hyperfibrinolytic role by inhibiting plasminogen activator inhibitor. Thus, activated protein C accounts for the hypocoagulation and hyperfibrinolysis characteristics observed in some trauma patients.

The third hypothesis focuses on trauma-induced neuro-hormonal and endothelial responses [38, 39]. Tissue injury from trauma induces sympathoadrenal responses and catecholamine release. Circulating catecholamine damages the endothelial glycocalyx and converts the endothelial function from antithrombotic to prothrombotic for local hemostasis. There is also a counterbalance mechanism of the anticoagulation and fibrinolytic responses in the blood to prevent this local response from extending beyond injury sites. However, this counterbalance mechanism is amplified after severe trauma injury, resulting in hypocoagulation and hyperfibrinolysis observed in some trauma patients.

To date, debates and controversies remain in these hypotheses [7, 13, 40]. Nevertheless, traumatic injury and shock related hypoperfusion have been widely accepted as the two important initiators of the early coagulopathy following trauma [24, 32, 41]. The severity of the trauma and the duration of shock appear to be positively related to the severity of coagulation dysfunction.

### Resuscitation phase

The resuscitation phase covers the first few days (i.e., 24–48 h) after trauma injury. During this phase, metabolic acidosis and hypothermia may develop together with hemodilution from resuscitation fluids used to improve hemodynamics. These factors may further impair and amplify the already existing coagulopathy from the trauma injury [42–44].

#### Metabolic acidosis

Clinical acidosis is commonly observed in trauma patients due to hypo-perfusion from massive blood loss. Impaired clotting enzyme activities have demonstrated the effects of acidosis on coagulation. Acidotic trauma patients showed prolonged PT and aPTT and decreased coagulation factor levels. Quantitatively, when pH was reduced from 7.4 to 7.0 *in vitro*, the activities of factor VIIa and factor VIIa/TF on phospholipid vessels decreased by more than 90 and 60%, respectively [45]. When pH was reduced from 7.4 to 7.1 in pigs, thrombin generation decreased to 47% of the control values [46]. In thrombin generation kinetics, acidosis moderately inhibited the initiation phase of thrombin generation, but persistently and dramatically inhibited the propagation phase [46]. These data showed that acidosis more severely inhibited the activation of factor V, VIII, IX, X and the formation of factor Xase and the prothrombinase complex in the propagation phase, compared to the activation of factor VIIa/tissue factor complex in the initiation phase [46].

The effects of acidosis on fibrinogen availability and metabolism were investigated in a swine model using stable isotope infusion and subsequent gas chromatograph mass spectrometry analysis [47]. Acidosis of pH 7.1 caused a 1.8-fold increase in fibrinogen breakdown rate compared to the control values but did not affect fibrinogen synthesis rate [47]. The accelerated consumption and unchanged production suggest a deficit of fibrinogen availability and support the supplementation of exogenous fibrinogen to improve hemostasis.

To restore coagulation function impaired by acidosis, bicarbonate solution was used to neutralize pH in a swine model after the induction of acidosis [48]. Acidosis of pH 7.1 depleted fibrinogen levels and platelets counts and impaired thrombin generation, clotting speed and clot strength [48]. The infusion of bicarbonate solution immediately corrected pH to 7.4. However, bicarbonate pH neutralization did not immediately recover the depleted substrate levels or coagulation dysfunction. Similar findings were observed when a different pH neutralizer, tris-hydroxymethyl-aminomethane, was used [49]. These findings demonstrated that acidosis-induced coagulopathy, once developed, cannot be corrected immediately by pH neutralization. Thus, the clinical focus of acidosis-induced coagulopathy should be on prevention instead of correction.

#### Hypothermia

The effects of hypothermia on the coagulation process have been estimated by cold-induced changes in standard clinical tests. Prolonged PT and aPTT have been shown in hypothermic patients and experimental animals, as well as plasma cooled *in vitro* [50–53]. The *in vivo* effects of temperature on thrombin generation kinetics were investigated in a swine model [46]. Hypothermia of 32 °C primarily inhibited the initiation phase of thrombin generation, involving the formation of factor VII/tissue factor complex [46]. The thrombin generation propagation phase, however, was not affected. Thus, compared to those observed in acidosis, hypothermia impairs the thrombin generation kinetics differently from acidosis.

Temperature effects on fibrinogen metabolism and availability were investigated in pigs with stable isotope infusion [54]. Hypothermia of 32 °C decreased fibrinogen synthesis rate by 50% of the control values, but fibrinogen breakdown rate remained unchanged [54]. Compared to the accelerated breakdown and unchanged synthesis by acidosis, hypothermia affects fibrinogen metabolism *via* different mechanisms. However, the decreased production and unchanged consumption by hypothermia indicate a similar outcome as acidosis: a potential deficit in fibrinogen availability.

#### Resuscitation

Following blood loss, fluid resuscitation is a routine clinical practice to restore tissue perfusion and hemodynamics. A variety of resuscitation fluids have been used around the world, with selections depending on availability, cost, and local clinical experience. Crystalloids, such as normal saline and lactated Ringer’s (LR) solution, are inexpensive and have been widely used for resuscitation [55–57]. Normal saline is a NaCl salt solution with an average pH of 5.0. LR has an average pH of 6.5 and has similar electrolytes to plasma, thus is considered as a more physiologically compatible fluid. In comparative trials of LR and normal saline in patients undergoing kidney transplant or aortic aneurysm repair, similar clinical outcomes of ICU stay, ventilation time, and complication incidence were observed in patients resuscitated with LR or normal saline, although patients with normal saline were more acidotic. In a rat model with moderate hemorrhage (36% of estimated total blood volume) and simultaneous resuscitation, normal saline and LR had equivalent survival rates [58]. However, LR resuscitation resulted in better survival after a massive hemorrhage (218% of estimated total blood volume) [58]. In a large animal model with femur fracture and 60% hemorrhage, normal saline and LR have similar effects on hemodynamics, oxygen metabolism and coagulation [59]. Normal saline required a larger resuscitation volume and was associated with poor acid base status and elevated serum potassium [59].

Colloids are highly effective at increasing the intravascular volume with a small volume increase in the interstitial space, compared with crystalloids. This volume-expanding advantage is logistically important in pre-hospital circumstances and in far forward battlefield conditions. Different colloids, such as albumin, gelatin, and hydrozyethyl starch, have been used clinically [60–63]. Although positive clinical outcomes have been reported in some clinical trials and animal studies, colloid resuscitation has been associated with a reduction in coagulation factors, platelet dysfunction and hemorrhagic complications [64–66]. In a swine model with traumatic hemorrhage, Hextend resuscitation caused severe reductions in coagulation factors, platelet counts and fibrinogen levels and impaired coagulation based on TEG. Those deteriorations persisted for the entire 6-h experimental duration,, whereas coagulation was restored 3 h after LR resuscitation [59].

With the emphasis on limiting crystalloids and increasing blood products, damage control resuscitation has been increasingly recognized and implemented in trauma care over the past decade [67–69]. Blood products, such as fresh frozen plasma (FFP), packed red blood cells (PRBC) and platelets, have been used for hemostatic resuscitation and hemodynamic resuscitation. As a proactive approach in damage control resuscitation, massive transfusion protocols quickly provide large amounts of blood products to critically injured and bleeding patients [70]. The selection and the order of the infusion of blood products in bleeding patients vary at different trauma centers [71, 72]. In both military and civilian trauma reports, higher ratios of plasma and platelets to PRBC appear to be more beneficial with improved survival [73, 74]. However, the use of blood components is also associated with increased risks in infection and organ failure [75–77]. The optimum ratios and doses of those blood products are still debatable.

Pharmaceutical hemostatic agents, such as fibrinogen concentrate, have been used as resuscitation to replenish fibrinogen levels. Among coagulation factors depleted after traumatic injury, fibrinogen is the first to drop to a critical level [47, 54, 78]. These findings support the notion of supplementing exogenous fibrinogen to restore coagulation function. The clinical use of fibrinogen concentrate has been shown in surgical patients to be efficacious, with improved clotting function and reduced transfusion requirements [79–83]. Large prospective clinical trials are on-going to investigate the efficacy of fibrinogen concentrate pre-hospital and in-hospital use in trauma patients.

### Late post-trauma phase

During the late post-trauma phase, the systemic levels of cytokines and hormones increase, leading to endothelial cell activation. The activated endothelial cells, circulating cytokines and thrombin, lead to a slow transition of the endothelial cell phenotype from antithrombiotic to prothrombotic. The endothelial cell activation also down-regulates thrombomodulin and fibrinolysis. In addition, the fibrinogen levels increase several folds due to acute phase responses. Overall, the coagulation process at this phase becomes the prothrombotic state, predisposing patients to venous thromboembolism, which leads to patients requiring heparin or a newer anticoagulant drug.

## Coagulation complications in sepsis

Coagulopathy in sepsis appears to be similar to the prothrombotic state observed in the late phase of trauma, although it is much less studied compared to trauma. During sepsis, the coagulation cascade is activated by inflammatory cytokine release and tissue factor [84–86]. Although the primary source of tissue factor remains unclear, it plays a key role in the activation of the coagulation cascade, *via* the binding of factor VII and the production of factor Xa for thrombin generation [86]. Inflammation also releases platelet activation factor to activate platelets, providing a surface for thrombin generation. In addition, pro-inflammatory cytokines are upregulated and play an important role in the suppression of anticoagulation. The enhanced prothrombic state and inhibited anticoagulation contribute to hypercoagulopathy and the development of DIC in sepsis [87]. As the severity of sepsis progresses, the dysfunctional coagulation leads to microvascular thrombosis and multiple organ dysfunction syndrome [85, 87].

Widespread intravascular activation of the coagulation system is the hallmark of DIC from various pathophysiological insults, such as sepsis. There are some similarities between DIC and early traumatic coagulopathy, including depleted coagulation factors and increased fibrinolysis [27, 34]. However, histological examination did not show disseminated clot formation in trauma patients [88]. The underlying mechanisms contributing to the development of sepsis DIC and trauma-induced coagulopathy remain unclear.

## Conclusion

Coagulation complications after trauma have been considered to be attributed to hypothermia, acidosis and hemodilution from blood loss and resuscitation. Clinical findings over the last decade have expanded our knowledge of this topic to shortly after trauma injury. Hemostatic manifestations may be present at hospital admission in some severely injured trauma patients, with mortality 3 to 4 times higher than those without coagulation complications. This recognition has led to the use of new terminology and the generation of some hypotheses in the trauma community. However, the underlying mechanisms related to the development of coagulation complications after trauma remain unclear. Continuing research effort and large clinical trials are warranted to improve our understanding and to facilitate the search of effective treatments for coagulation complications after trauma.

## Abbreviations

ACT: Acute coagulopathy of trauma
aPTT: Activated partial thromboplastin time
ATC: Acute traumatic coagulopathy
DIC: Disseminated intravascular coagulation
FFP: Fresh frozen plasma
LR: Lactated Ringer’s
PAI: Plasminogen activator inhibitors
PRBC: Packed red blood cells
PT: Prothrombin time
ROTEM: Rotational thromboelastometry
TEG: Thromboelastography
TIC: Trauma induced coagulopathy
tPA: Tissue-type plasminogen activator
vWF: von Willebrand Factor.
